# Effect of miR-223-3p and miR-328a-3p Knockdown on Allergic Airway Inflammation in Rat Precision-Cut Lung Slices

**DOI:** 10.3390/cells14020104

**Published:** 2025-01-12

**Authors:** Joanna Nowakowska, Maria Kachel, Wojciech Langwiński, Kamil Ziarniak, Aleksandra Szczepankiewicz

**Affiliations:** 1 Molecular and Cell Biology Unit, Department of Pediatric Pulmonology, Allergy and Clinical Immunology, Poznan University of Medical Sciences, 60-572 Poznan, Poland; maria00kachel@gmail.com (M.K.); wlangwinski654@gmail.com (W.L.); kamilziarniak@gmail.com (K.Z.); 2 Doctoral School, Poznan University of Medical Sciences, 60-812 Poznan, Poland; 3 Centre of Experimental Medicine, Poznan University of Medical Sciences, 60-806 Poznan, Poland

**Keywords:** PCLS, miRNA, allergic inflammation

## Abstract

Asthma is a major non-communicable disease whose pathogenesis is still not fully elucidated. One of the asthma research models is precision-cut lung slices (PCLSs), and among the therapeutic options, miRNA molecules are of great interest. The aim of our study was to investigate whether inhibition of miR-223-3p and miR328a-3p affects the inflammatory response in PCLSs derived from a rat with HDM-induced allergic inflammation and a control rat. We generated rat PCLSs and transfected them with miR-223-3p and miR-328a-3p inhibitors. RNA was isolated from PCLSs and analyzed by qPCR. We also examined the proteins in the culture medium using the Magnetic Luminex Assay. The comparison between miRNA-transfected PCLSs and non-transfected controls showed significant differences in the expression of several genes associated with allergic inflammation, including *Il-33*, *Ccl5*, *Prg2* and *Tslp*, in both the rat with allergic inflammation and the control rat. In the culture medium, we found no significant differences in protein levels between rat with allergic inflammation and the control. Our study highlighted some important issues: the need to extend the model by including more biological replicates, the need to standardize culture conditions, and the need to consider co-transfection with several miRNA inhibitors when modifying miRNAs expression in the PCLS model.

## 1. Introduction

According to the World Health Organization, asthma is a major non-communicable disease affecting both children and adults [1]. The Global Burden of Disease Study estimated that 262 million people worldwide were affected by asthma in 2019, and it was the cause of death for 461,000 of them [2].

We can manage asthma, but its pathogenesis is still not fully understood. Various experiments have been conducted to explain the cause and the course of the disease, including clinical studies, in vivo experiments in animal models, and in vitro studies. Particularly predominant in the literature are studies on cell lines grown in monolayers, such as bronchial epithelial cell lines, which are the most popular [3,4]. A step forward was the introduction of an air–liquid interface (ALI) culture, which allows us to study the airway-specific environment, including mucociliary cell differentiation [5,6]. Unfortunately, in vitro studies are limited to one or two cell types and do not allow for the study of intercellular communication or the microenvironment in lung cells and tissues. Thus, a large number of studies have been conducted in animal models, usually rats or mice, which allow us to observe the response of the whole organism to the substances and processes we are studying in vivo [7,8]. 

Following the “4R” principle (*replacement, reduction, refinement, responsibility*), researchers are trying to limit the number of animals used in scientific experiments. Organotypic tissue models, such as precision-cut lung slices (PCLSs), appear to bridge the gap between cell lines and animal studies. PCLSs are tissue sections, cultured ex vivo, that preserve native microenvironment (cell–cell and cell–matrix interactions) of the lung and allow for the determination of the effect of delivered agents on the surrounding tissues. This model also reduces the number of animals—the lungs of animals from other experiments can be used to generate PCLSs, replacing an in vivo animal experiment with an ex vivo tissue study [9].

PCLSs can also provide a platform for drug testing [10]. Among the therapeutic targets, of great interest are microRNAs (miRNAs), which were discovered by Ambros and Ruvkun and were awarded the Nobel Prize in Physiology or Medicine in 2024 [11]. MicroRNAs (miRNAs) are small (about 22 nucleotides), non-coding RNA molecules that are involved in the regulation of gene expression at the post-transcriptional level [12]. Synthetic analogs (mimics) or inhibitors of miRNAs have emerged as promising therapeutic agents, with several molecules having already been tested in clinical trials [13]. Among these, miR-122 (miravirsen) is the most advanced [14].

While the key role of miRNA molecules has previously been established in the pathogenesis of asthma and allergic diseases [15], the search for a novel, clinically effective drug remains in its infancy. In the previous study, we identified two differentially expressed miRNAs in an in vivo rat model of asthma: increased expression of miR-223-3p and decreased expression of miR-328a-3p in the lungs of rats with HDM-induced allergic inflammation compared to control rats [16]. MiR-223-3p has emerged as one of the most studied and promising miRNAs in asthma. Its effects on specific signaling pathways (mostly inflammatory, such as NF-kβ, IL-1 signaling, but also cell proliferation, such as PI3K/Akt and mTOR), as well as its expression in various body fluids (blood, bronchoalveolar lavage, sputum, nasal lavage fluid), cells (bronchial epithelial, endothelial, smooth muscles, macrophages, neutrophils), and tissues (including lung), both human and animal (murine, rat, zebrafish), have been repeatedly analyzed [17,18,19]. The other miRNA, miR-328a-3p, has been linked to wound repair in the human bronchial epithelium [20]. Several studies have indicated its association with allergic inflammation, as seen in serum [21], exhaled breath condensate [22], and airway smooth muscle cells [23]. 

Therefore, the objective of this study was to determine whether inhibition of miR-223-3p and miR328a-3p would affect the inflammatory response in PCLSs derived from a rat with HDM-induced allergic inflammation and a control rat that were transfected with inhibitors for miR-223-3p and miR-328a-3p. 

## 2. Materials and Methods

### 2.1. Animals, PCLS Generation, and Transfection

#### 2.1.1. Rats

Lung tissue was obtained from Brown Norway male rats (BN/Crl) with body masses ranging between 90 and 130 g, which were purchased from Charles River Laboratories (Sulzfeld, Germany). One rat with allergic inflammation and one control rat were used. All experimental procedures were conducted according to European and Polish legislation and were approved by the Local Ethical Committee for the Experiments on Animals (approval numbers 75/2021, 21/2023, and 22/2023), Poznan University of Life Sciences (Poznan, Poland). Following the 4Rs principle, we used tissues from animals scheduled for euthanasia in another experiment. Rats were sacrificed by decapitation. 

#### 2.1.2. Rat Model of Allergic Inflammation

We induced allergic inflammation in rats using treatment with house-dust mite extract (HDM). Animals were subcutaneously (s.c.) injected with 45 µg of HDM (Citeq Biologics, Groningen, The Netherlands) in 250 µL Al(OH)_3_ once a week for three weeks; then, they were intranasally challenged with 120 µg of HDM in 50 µL of 0.9% NaCl (i.e., 25 µL to each nostril) three times a week for four weeks. The control group received Al(OH)_3_ for s.c. injections and 0.9% NaCl intranasally. 

#### 2.1.3. Histological Staining

Lung tissues were fixed in 10% neutral-buffered formalin for 72 h. Then, the tissues were dehydrated with ethanol solutions (70%, 80%, 95%, 100%), embedded in paraffin, and cut into 2.5 μm sections. To remove paraffin, sections were incubated in a heater, placed in xylene, hydrated using decreasing ethanol concentrations, and then stained with hematoxylin and eosin (H+E), dehydrated, and cover-slipped. Representative photographs were captured using an Axio Lab 1 microscope (Carl Zeiss, Jena, Germany). Eosinophilic infiltration in the airways after histological staining was assessed by counting eosinophils from 10 different fields of view of specimens from allergic and control rats, which was conducted by a veterinary pathologist. Airway smooth muscle thickness was assessed by a veterinary pathologist in 5 random fields of view using ZEN 2.3 (blue edition) software (Carl Zeiss Microscopy GmbH, Oberkochen, Germany, 2011).

#### 2.1.4. PCLS Generation

Subsequent to the sacrifice of the animal, the lungs were filled with low-melting agarose. Once the agarose had solidified, we excised the lungs from the animal and cut them into fragments using a scalpel. Then, the fragments were attached to the vibratome disc (Leica VT 1000S (Leica Biosystems, Nussloch, Germany)) and sliced into 300 µm fragments. Next, PCLSs were prepared from these slices using a 4mm diameter biopsy punch. The detailed protocol for the PCLS preparation is available in our previous publication [24]. The ciliary beating was assessed before and after transfection to confirm PCLS viability using a Nikon Ts2R microscope equipped with a high-speed camera (Andor Zyla 4.2).

#### 2.1.5. PCLS Transfection

Transfection was performed with INTERFERin transfection reagent (Polyplus transfection, Illkirch, France) according to the manufacturer’s recommendation. The transfection mixtures were prepared in OptiMem (Thermo Fisher Scientific, Waltham, MA, USA). A complete culture medium was added to each well to obtain a total volume of 100 µL. Each experiment included both untreated samples (marked as CTRL) and mock control samples (transfection reagent without miRNA). For all experiments, a custom-synthesized scrambled miRNA molecule conjugated to a far-red dye, TYE-665, at the 3’ end (5′ ACGTCTATACGCCCA 3′TYE665) was used (Qiagen, Hilden, Germany) as a negative control. For the transfection with inhibitors of miR-223-3p (5′ GGGGTATTTGACAAACTGAC 3′) and miR-328a-3p (5′ GGAAGGGCAGAGAGGGCCA 3’), we used unlabeled molecules at a concentration of 50 nM (miRCURY LNA miRNA inhibitor, Qiagen). The optimal concentrations of transfection reagent and inhibitors for miRNAs were optimized in our previous study [24].

### 2.2. Lactate Dehydrogenase Assay (LDH)

Lactate dehydrogenase activity (Cytotoxicity detection kit LDH, Sigma-Aldrich, St. Louis, MO, USA) was assessed daily in secreted supernatants, according to the manufacturer’s protocol. Prior to use, the collected supernatants were diluted in DMEM-F12 medium (60:40 *v*/*v*). Absorbance at 490 nm was measured using a microplate reader (ASYS UVM 340 (Biocompare, South San Francisco, CA, USA)), with 620 nm as a reference, against a background control (i.e., DMEM-F12 medium). Separate PCLSs were treated with a 2% Triton X-100 solution for 30 min to induce total cell lysis and maximal LDH release. The data are presented as relative absorbances at each time point and for individual PCLS.

### 2.3. Fluorescent Microscope Imaging

Rat PCLSs were imaged in real time before and after transfection using a 4× objective. A conventional inverted fluorescence microscope (Nikon Ts2R (Nikon Instruments, New York, NY 11747, USA)) was used, employing the brightfield and red fluorescence (Cy5) channels. The method of fluorescence reading and comparison with non-transfected controls was the approach we used in earlier studies [24].

The specific microscope settings are detailed in the corresponding figure legends. 

### 2.4. RNA Analysis

Forty-eight hours after transfection, each PCLS was placed in 200 µL of Qiazol (Qiagen) and stored at −80 °C. The RNA was isolated from each PCLS separately according to a modification of the phenol–chloroform isolation method developed by Langwiński et al. [25].

For reverse transcription, 100 ng of RNA was used along with the GoScript Reverse Transcription Kit (Promega, Madison, WI, USA). The relative expression analysis was conducted using quantitative reverse transcription PCR (qRT-PCR) and GoTaq qPCR Master Mix (Promega), employing specific primers for the following genes: *Il-4*, *Il-5*, *Il-6*, *Il-13*, *Il-33*, *Tnfα*, *Tslp*, *Gm-csf*, *Prg2*, *Muc*, *Ccl5*, *Ccl24*, *Ccl26*, *Tgfβ1*, and *Epx* (Supplementary Table S1). Calculations were performed using the delta–delta Ct method, with *Gapdh* serving as the reference gene.

### 2.5. Protein Analysis

#### 2.5.1. Protein Isolation and Western Blotting

Forty-eight hours post-transfection, each PCLS was placed in 50 µL of RIPA buffer (Sigma-Aldrich), which had been supplemented with protease and phosphatase inhibitor cocktails (Roche, Basel, Switzerland). The samples were sonicated in the same way as samples intended for RNA isolation. After that, samples were centrifugated at 4 °C for 15 min. The resulting supernatants were then collected for further analysis.

Western blotting was then performed using 5 μg of each protein sample according to the manufacturer’s instructions, with precast mini 14% gels. Electrophoresis was conducted using the Mini Gel Tank system at 220 V and the iBlot transfer system (P0 program; Thermo Fisher Scientific). The membranes were blocked in 1% bovine serum albumin (BSA) solution for 1 h and incubated overnight at 4 °C with the primary antibody in TBST supplemented with 1% BSA (dilution 1:1000). In all reactions, the secondary antibody used was goat anti-rabbit IgG HRP antibody HAF008 (dilution 1:1000). Instead of β-actin detection, total protein normalization was performed according to the manufacturer’s protocol (No-Stain Protein Labeling Reagent, Thermo Fisher Scientific). Signals were then visualized by chemiluminescence using SuperSignal West Pico PLUS Chemiluminescent Substrate (Thermo Fisher Scientific). Images were captured by the ChemiDoc Imaging System (BioRad Laboratories, Munich, Germany). The intensity of the bands was quantified using ImageJ software [26].

#### 2.5.2. Measurement of Proteins Secreted by PCLSs to the Culture Medium

The culture medium was collected daily from each well individually and frozen at −80 °C for further analysis. The concentration of proteins in the medium, which had been diluted 50:50 with Calibrator Diluent RD6-52, was measured using a Luminex MAGPIX analyzer (Thermo Fisher Scientific) and a 14-plex Rat Magnetic Luminex Assay (catalog no. LXSARM-14, R&D Systems, Minneapolis, MN, USA), according to the manufacturer’s protocol. The analytes selected for the assessment included the following proteins: CXCL2, ICAM-1, IL-1α, IL-2, IL-6, IL-13, TNF*α*, GM-CSF, IFNγ, IL-1*β*, IL-4, IL-10, IL-18, and VEGF. The xPONENT 4.1 software was applied for the acquisition and assay design.

### 2.6. Statistics

Statistical analyses were performed using PQstat software (v.1.8.6). In all analyses, the normality of the distribution was tested using the Lilliefors test. All study variables were independent, so for comparisons between two groups, Student’s *t*-tests for independent groups were performed. For comparisons between more than two groups, one-way analysis of variance (ANOVA) for independent groups was performed. The homogeneity of variance was checked using the Fisher–Snedecor test. For groups that did not show equal variances, the *t*-Student test was performed with the Cochran–Cox correction. A value of *p* < 0.05 was considered statistically significant. According to the order of magnitude for *p*-values, analyses were marked with asterisks (* for *p* < 0.05, ** for *p* < 0.005, *** for *p* < 0.0005).

## 3. Results

### 3.1. Histological Confirmation of Allergic Inflammation in Rats

**Figure 1:**
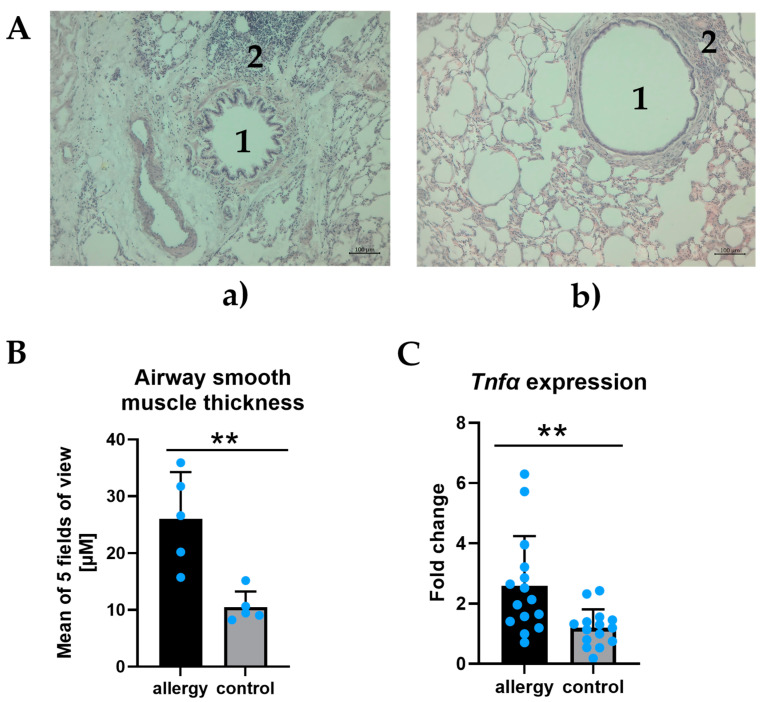
(**A**)—Hematoxylin and eosin staining of lung tissues in allergic inflammation rat (**a**) and control rat (**b**). In the allergic inflammation rat, lung airway obstruction (1) and inflammatory cell infiltration (2) were observed when compared to the control rat. Pictures were captured at 10× magnification. (**B**)—Airway smooth muscle thickness from 5 random fields of view, assessed for the rat with allergic inflammation (n = 5) and the control rat (n = 5). Unpaired *t*-test (*p* = 0.003); error bars represent standard deviation (SD). (**C**)—*Tnfα* expression in PCLS-derived RNA samples from the rat with allergic inflammation (n = 15) and the healthy rat (n = 15), normalized to *Gapdh*. Unpaired *t*-test with Welch’s correction (*p* = 0.006); error bars represent SD. According to the order of magnitude for *p*-values, analyses were marked with asterisks (** for *p* < 0.005).

Allergic lungs presented inflammatory cell infiltration and airway obstruction (Figure 1A). To further confirm allergic inflammation, we also assessed the number of eosinophils per 10 fields of view for each specimen (mean number 12.6 for the rat with allergic inflammation, compared with mean number 3.9 for the control rat), as well as the airway smooth muscle thickness, which was significantly higher in the rat with allergic inflammation compared to the control one (Figure 1B) Allergic inflammation was also confirmed in the gene expression from the PCLSs. We observed significantly higher expression of the *Tnfα* in the PCLS from the rat with allergic inflammation compared to the PCLS from the control rat (Figure 1C) 

### 3.2. Analysis of Transfection Efficacy and Lack of Cytotoxicity

**Figure 2:**
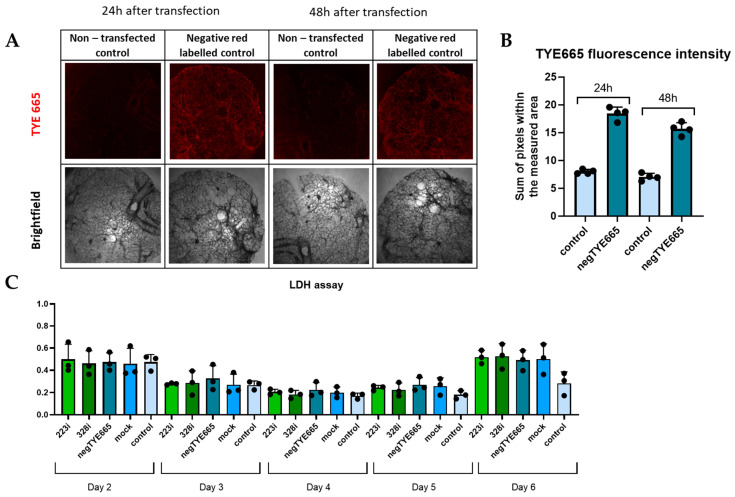
(**A**)—Representative bright- and widefield fluorescent microscopy 24 h and 48 h post-transfection, 4× objective, TYE 665 dye (laser: 15, exposure time: 200 ms—24 h and 120 ms—48 h). (**B**)—Sum of TYE 665 fluorescence intensity; each bar represents mean value ± SD (n = 4 regions). (**C**)—Representative LDH release in the culture medium on days 2–6; each bar represents mean value ± SD (n = 3).

To transfect PCLS with miRNA, we used the method we developed and previously described [24]. Transfection was performed using an INTERFERin transfection reagent according to the manufacturer’s recommendation. Each experiment included untreated samples (marked as CTRL); mock control samples (i.e., transfection reagent without miRNA); and a custom-synthesized scrambled miRNA molecule conjugated to a far-red dye, TYE-665, at the 3’ end (marked as red labeled negative control/TYE665). For the transfection with inhibitors, we used rno-miR-223-3p and rno-miR-328a-3p at a concentration of 50 nM. We confirmed the transfection in PCLSs transfected with negative control labeled with TYE-665 (red signal) as compared to control PCLSs that were not transfected (Figure 2A,B). Subsequent analysis using the LDH assay confirmed that miRNA transfection did not cause cytotoxicity in PCLSs over the course of the experiment (days 2–6) (Figure 2C)

### 3.3. Igf1R: A Target Gene for Both miR-223-3p and miR-328a-3p

**Figure 3:**
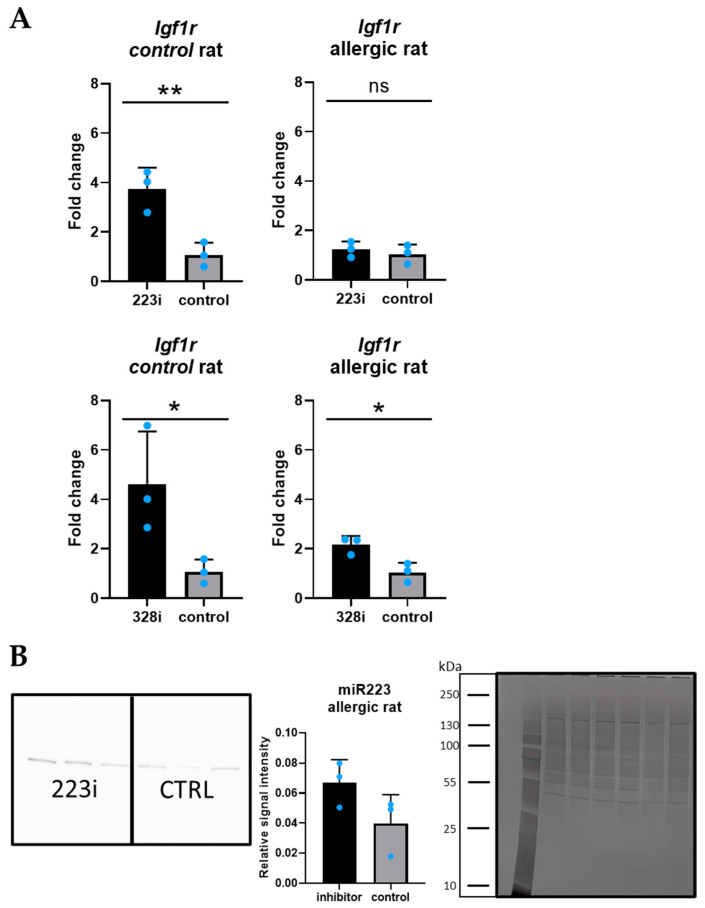
(**A**)—The results of the qPCR analysis of Igf1r gene in PCLS from the control rat (*p* = 0.009) and PCLS from the rat with allergic inflammation (*p* > 0.05) for the miR-223 inhibitor (comparison between inhibitor-transfected and non-transfected PCLS) and the miR-328a inhibitor (control rat: *p* = 0.048; rat with allergic inflammation: *p* = 0.021); all results were normalized to Gapdh. Unpaired *t*-test, each bar represents mean value ± SD (n = 3). (**B**)—Representative image of Western blot result (miR-223-3p, rat with allergic inflammation) with relative signal intensity measured in ImageJ (v2.0.0-beta4), approximate marker and total protein normalization; each bar represents the mean value ± SD (n = 3). (ns = non-significant, * for *p* < 0.05, ** for *p* < 0.005).

We selected *Igf1R* as the target gene for both miR-223 and miR-328a based on miRTarBase [27] and the literature [28,29]. In qPCR, significantly higher expression of the target gene was observed for inhibitors of both miRNA molecules compared to untransfected controls in the PCLS from the healthy rat (Figure 3A). In the PCLS from the rat with allergic inflammation, *Igf1R* expression was significantly increased for miR-328a-3p, but not for the miR-223-3p inhibitor (Figure 3A). At the protein level (Western blot), we did not see any significant changes between the inhibitor and non-transfected groups for PCLSs derived from both the rat with allergic inflammation and the control rat transfected with miRNA-223-3p, as well as with miRNA-328a-3p (Figure 3B).

### 3.4. Analysis of Culture Medium—Panel of Cytokines Associated with Allergic Inflammation

Of the 14 proteins analyzed in the PCLS culture medium, nine (i.e., CXCL2, ICAM-1, IFNγ, IL-10, IL-13, IL-18, IL-2, IL-6, and VEGF) showed concentrations above the detection threshold. The reaction was performed according to the manufacturer’s recommendations. The reader was set to read a minimum of 50 beads with a unique fluorescent signature per region with the output of median fluorescence intensity per sample, and all standards were within the detectable range (Supplementary Figure S1). We found no significant differences in the protein levels in the culture medium from the PCLS from the allergic inflammation rat as compared to the control rat (Table 1).

### 3.5. The Effect of Silencing miR223-3p and miR328a-3p Expression on Genes Associated with Allergic Inflammation in PCLSs from the Rat with Allergic Inflammation and the Control Rat

**Figure 4:**
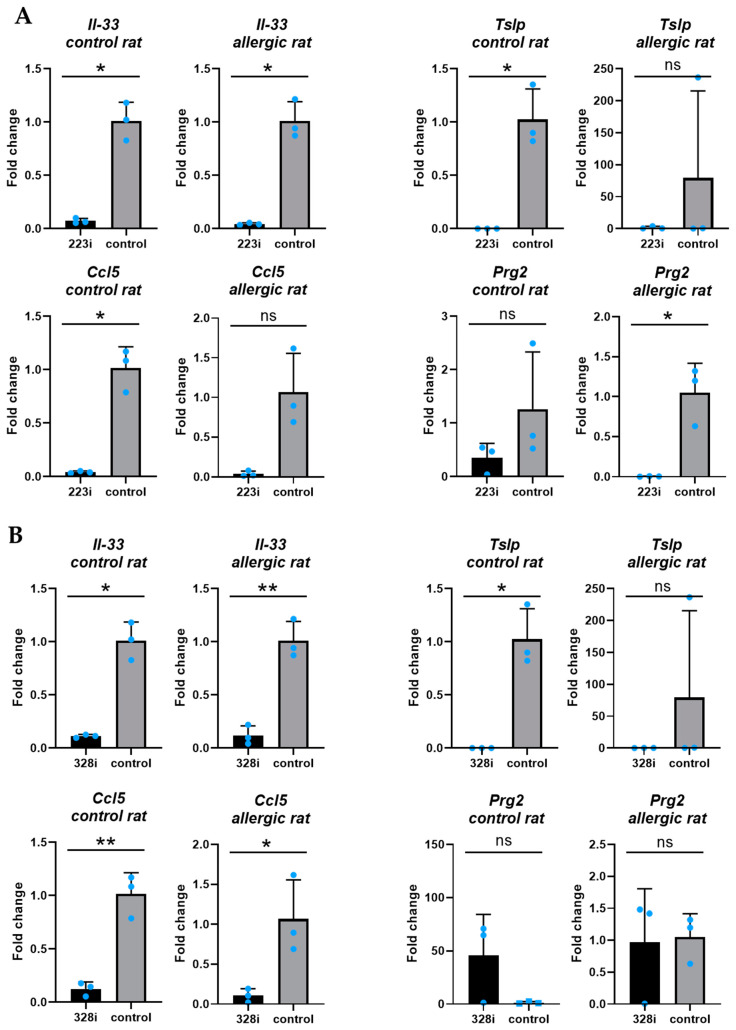
(**A**)—Comparative expression analysis between PCLSs transfected with miR-223 inhibitor (223i) and non-transfected (control) PCLSs from the control rat and the rat with allergic inflammation for the following genes: Il-33_control rat (*p* = 0.011), Il-33_allergic rat (*p* = 0.011), Tslp_control rat (*p* = 0.025), Tslp_allergic rat (*p* > 0.05), Ccl5_control rat (*p* = 0.014), Ccl5_allergic rat (*p* > 0.05), Prg2_control rat (*p* > 0.05), and Prg2_allergic rat (*p*=0.039). All results were normalized to Gapdh; unpaired *t*-test/unpaired *t*-test with Welch’s correction; each bar represents mean value ± SD (n = 3). (**B**)—Comparative expression analysis between PCLSs transfected with miR-328a inhibitor (328i) and non-transfected (control) PCLSs from the control rat and the rat with allergic inflammation for the following genes: Il-33_control rat (*p* = 0.012), Il-33_allergic rat (*p* = 0.002), Tslp_control rat (*p* = 0.025), Tslp_allergic rat (*p* > 0.05), Ccl5_control rat (*p* = 0.002), Ccl5_allergic rat (*p* = 0.028), Prg2_control rat (*p* > 0.05), and Prg2_allergic rat (*p* > 0.05). All results were normalized to Gapdh; unpaired *t*-test/unpaired *t*-test with Welch’s correction; each bar represents mean value ± SD (n = 3). According to the order of magnitude for *p*-values, analyses were marked with asterisks (ns = non-significant, * for *p* < 0.05, ** for *p* < 0.005).

The analysis of genes associated with allergic inflammation (*Il-4, Il-5, Il-6, Il-13, Il-33, Tnfα, Tslp, Gm-csf, Prg2, Muc, Ccl5, Ccl24, Ccl26, Tgfβ1*, and *Epx*) in PCLSs transfected with miR-223 inhibitor (223i) compared to non-transfected PCLSs (control) showed that the expression levels of *Il-33, Tslp, and Ccl5* were significantly decreased in the control rat. For the rat with allergic inflammation (allergic rat), the expression levels of *Il-33* and *Prg2* were significantly decreased in the PCLS transfected with the miR-223 inhibitor (223i) compared to the non-transfected PCLS (control) (Figure 4A). 

The analysis of genes associated with allergic inflammation (*Il-4, Il-5, Il-6, Il-13, Il-33, Tnfα, Tslp, Gm-csf, Prg2, Muc, Ccl5, Ccl24, Ccl26, Tgfβ1,* and *Epx*) in PCLS transfected with miR-328a inhibitor (328i) compared to non-transfected PCLS (control) showed that the expression levels of *Il-33, Tslp*, and *Ccl5* were significantly decreased in the control rat. For the rat with allergic inflammation (allergic rat), the expression levels of *Il-33* and *Ccl5* were significantly decreased in PCLS transfected with miR-328a inhibitor (328i) compared to non-transfected PCLS (control) (Figure 4B). 

## 4. Discussion

This is the first study to analyze allergic inflammation-derived rat PCLSs with regard to the modification of miRNA expression. In this study, we used our previously developed method of PCLS transfection [24] with miRNA inhibitors to analyze the effect of silencing specific miRNAs, miR-223-3p and miR-328a-3p, in lung tissue slices derived from a control rat as well as a rat with HDM-induced allergic airway inflammation. This study also marks the first time we have used RNA isolated from a single PCLS rather than combining RNA from several eluates, according to the method developed recently by Langwinski et al. [25]

A similar research model was previously applied by Danov et al. [30] in mice sensitized with HDM extract. These researchers analyzed PCLS derived from a sensitized and a control mouse infected with rhinovirus. The authors then compared the panel of allergic cytokines between HDM-sensitized and non-sensitized PCLSs using ELISA and observed significant differences in the concentrations of several interleukins, including IL-4, IL-5, IL-10, and IL-33. What differentiates this study from our work is the time of the collection of the medium. In our study, we analyzed the medium 48 h after transfection, the equivalent of five days of culture, while Danov et al. analyzed the medium after 48 h of culture. The authors of the study, however, did not provide information regarding the specific plates used or what volume of medium was utilized for PCLS culture. Moreover, two PCLS per well were used for analysis, which might have affected the concentration of pro-inflammatory cytokines.

In the context of allergic airway inflammation, the most extensively studied models so far have been mouse-derived PCLSs. In the study by Kortekaas et al. [31], an exacerbation cocktail (IL-1β, IL-6, KC/CXCL1, and TNFα) was incubated with mouse PCLSs for 48 h, and the expression of epithelial genes was tested, including the MUC5AC gene, but no significant changes were observed between the treatment and control groups. However, it is difficult to assess to what extent this result could have been influenced by the automated RNA extraction method (which is usually associated with decreased quality of the material as compared to manual methods). Moreover, the study used PCLSs cultured in 12-well plates, yet there was no information on the number of PCLSs per well or the amount of culture medium utilized. 

Alvarez-Simon et al. [32] stimulated mouse PCLSs ex vivo with a solution of HDM extract for 24 h and observed increased TSLP and IL-33 production in the culture medium (using ELISA). In the present study, we also observed differences in *Tslp* and *Il-33* gene expression in groups treated with inhibitors. However, these differences were not observed in proteins secreted into the culture medium. It should be emphasized that, for these measurements, we used conditioned media from a single PCLS cultured per well. 

In regard to studies performed on human PCLSs, Stephenson et al. [33] stimulated PCLSs (derived from a healthy donor as well as a donor with idiopathic pulmonary fibrosis) with IL-33 or TGF*β* and then tested whether this combination would affect lung fibrosis-related gene expression. They did not observe significant changes in gene expression in the experimental model; however, it should be emphasized that the researchers pooled four different PCLSs for RNA extraction and expression analysis, and that could have influenced the results.

In a comparative analysis of the studies using PCLSs across diverse species, we observed that culture conditions are crucial for the interpretation of the results. This includes the concentration of agarose, the thickness of the scrapings, the format of the plates (12-well, 24-well, or 96-well plates; most studies lack this information), and the number of PCLSs per well, along with the volume of the medium. Thus, standardizing the number of PCLSs and the amount of medium per well is essential to allow for future comparisons of gene expression and protein levels in the medium between different models.

As for the miRNA transfection experiment, we observed a significant effect of silencing miRNA on the target gene’s mRNA expression level, as well as the expression of genes related to allergic inflammation (e.g., *Il-33* or *Tslp*), but the changes at the protein level in the culture medium were less evident. Therefore, we can conclude that the miRNA knockdown influenced the expression on the mRNA level inside the cells and that qPCR was more sensitive in detecting changes in the expression, whereas the Luminex assay may not be sufficiently sensitive to detect subtle changes in the protein expression.

The main limitation of the present study is the limited sample size (n = 3 PCLS samples per condition) and the high variability between biological replicates, as depicted in the bar plots for the PCLS data in our study. This variability is also present in the previous studies that we discussed above. Despite the fact that the PCLSs used in our study were derived from the same animal and the same region of the lungs and were cultured in standardized conditions, we observed substantial heterogeneity. The analysis of a large amount of available data indicates that PCLSs show large heterogeneity despite standardized culture conditions, and this makes it difficult to draw conclusions from a limited sample size. To address these challenges, we recommend that future studies using PCLSs should include a larger number of biological replicates per condition tested, and we discourage other researchers from pooling different PCLS samples into one RNA extraction to avoid compensatory effects between PCLSs. The use of a single animal per experimental group was intended to reduce the number of animals used for this type of study and to replace the animals with PCLSs. 

The main limitation in the number of PCLSs generated in this case was the time between animal sacrifice and the start of culture, which necessitated optimization to ensure the quality of the culture material.

## 5. Conclusions

The analysis of the effect of miRNA inhibitors on allergic inflammation parameters in PCLSs revealed reduced the expression of pro-inflammatory cytokines in samples treated with an miRNA inhibitor compared to the non-transfected group. This was observed in both the rat with induced allergic airway inflammation and the control rat, thus suggesting this approach as a potential therapeutic strategy. However, given the substantial variability between PCLSs from a single experimental animal, it is difficult to draw definitive conclusions. Our study showed that experiments using PCLS require the inclusion of more biological replicates as well as the standardization of PCLS culture conditions, irrespective of the species. The results of our transfection study also indicate that, in the case of modifying miRNA expression, co-transfection with several miRNA inhibitors could amplify the observed biological effects in cultured PCLS.

## Figures and Tables

**Table 1:** Mean concentrations of allergic inflammation cytokines in culture medium from PCLSs from the rat with allergic inflammation and the control rat (pg/mL).

Rat Protein Name	Mean Protein Level ± SD (Rat with Allergic Inflammation)	Mean Protein Level ± SD (Control Rat)
CXCL2	1.95 ± 0.58	1.76 ± 0.73
ICAM-1	5.91 ± 1.22	5.80 ± 2.34
IFNγ	0.96 ± 1.82	0.32 ± 0.95
IL-10	0.05 ± 0.06	0.04 ± 0.07
IL-13	0.04 ± 0.01	0.04 ± 0.02
IL-18	0.17 ± 0.03	0.22 ± 0.07
IL-2	0.08 ± 0.04	0.07 ± 0.05
IL-6	8.49 ± 3.45	7.62 ± 4.08
VEGF	0.78 ± 0.45	0.72 ± 0.60

## Data Availability

All raw and processed data will be made available upon reasonable request to the corresponding author.

## Supplementary Materials

The following supporting information can be downloaded at: https://www.mdpi.com/article/10.3390/cells14020104/s1, Table S1. qPCR rat primers sequences; Figure S1. Luminex assay performance for 14-cytokine plex from conditioned medium from PCLS transfected with miR-223-3p and miR-328a-3p inhibitors and non-transfected controls using MAGPIX.
