# Supplementary material for: Effect of miR-223-3p and miR-328a-3p Knockdown on Allergic Airway Inflammation in Rat Precision-Cut Lung Slices
Source: Cells. 2025 Jan 12;14(2):104. doi: 10.3390/cells14020104 (PMC11763956; doi:10.3390/cells14020104)
Supplement: Supplementary file 1 [file cells-14-00104-s001.zip › cells-3354557-supplementary.pdf]

Supplementary files

Supplementary Table S1. qPCR rat primers sequences

Rat gene name	Primer F (5′-3′)	Primer R (5′-3′)
Il-4	TACCAGGAGCCATATCCACGGATG	TGTGGTGTTCTTCGTTGCTGTGAG
Il-5	GTTGACGAGCAATGAGACGAT	GCCACACTTCTCTTTTTGTCC
Il-6	CTCGAGCCCACCAGGAACGA	TGGCTGGAAGTCTCTTGCGGA
Il-13	CTCGCTTGCCTTGGTGGTCT	TGCACAGGGAAGTCTTCTGGT
Il-33	TGAGTATCCAAGGAACTTCACTGCT	TGGTCGTTGTATGTGCTCAGGG
Tnfα	AGAACTCCAGGCGGTGTCT	GAGCCCATTTGGGAACTTCT
Tslp	GCCCTGCAGCAAACCAGTCT	TGGACCCCAGGACTATGCTCT
Gm-csf	TTCTCCATCCAGAGGCCGAC	GGTGAGGTTGCCCCGTAGAC
Prg2 (Mbp – major basic protein)	CACAACGAGTCTGATGGGTGA	CCGGCAGACCAACTGAGCTT
Muc2ac	GGGCACGTGAATGGACAGGTG	TATGTGCAGCCCAAGGCGTG
Ccl5 (Rantes)	GCATCCCTCACCGTCATCCTC	GCACTTGCTGCTGGTGTAAAA
Ccl24	CCCTCATCTTGCTGCGTGAC	CCCTTTTTAGTGATGAAGATGACCCC
Ccl26	GGTGTTGCCACGTGTGAGAGT	CATTGGGGCAGGAGCTGTCG
Tgfβ1	TGAGTGGCTGTCTTTTGACG	CAGGAAGGGTCGGTTCATGT
Epx (Epo – eosinophil peroxidase)	GACCACGGGCTTCCAGGATA	TCAATGTTGTCGGGCGTCTT
Gapdh	AACTCCCTCAAGATTGTCAGCAA	GGCATGGACTGTGGTCATGA

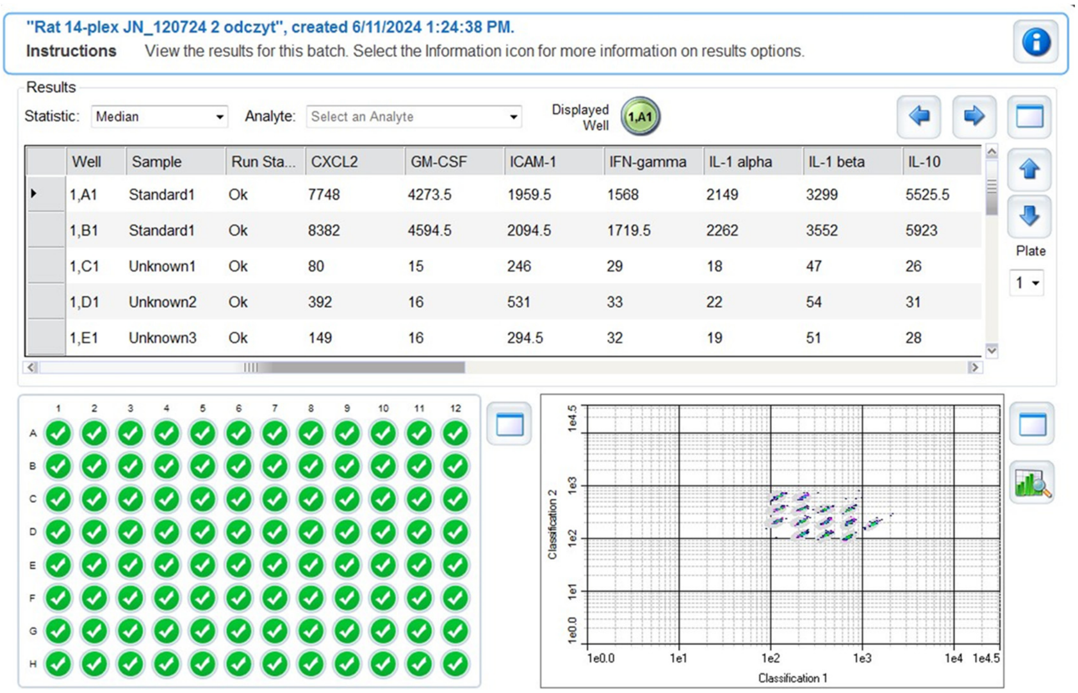

Supplementary Figure S1. Luminex assay performance for 14-cytokine plex from conditioned medium from PCLS transfected with miR-223-3p and miR-328a-3p inhibitors and non-transfected controls using MAGPIX
